# Designing multiple charge carrier separation pathways in core-type near infrared colloidal nanocrystal for broadband photodetector†

**DOI:** 10.1039/d4ra08792e

**Published:** 2025-02-27

**Authors:** Hyunjung Kim, Yoonji Jeong, Wan-Gil Jung, Minju Kim, Jiyoon Yang, Minseo Kim, Yeonsu Han, Hyun Ko, Sung Won Hwang, Myeong Jin Kim, Jong Woo Lee, Won-Jin Moon, Hanleem Lee

**Affiliations:** a Research Institute of Basic Sciences, Sungkyunkwan University 2066 Seobu-Ro Suwon 16419 Republic of Korea; b Department of Chemistry, Sungkyunkwan University 2066 Seobu-Ro Suwon 16419 Republic of Korea; c Department of Chemistry, Myongji University 116 Myongji Ro Yongin Gyeonggi-do 17058 South Korea hanleem@mju.ac.kr; d Institute for Quantum Biophysics (IQB), Sungkyunkwan University 2066 Seobu-Ro Suwon 16419 Republic of Korea; e Korea Basic Science Institute, Gwangju Center 77 Yongbong-ro, Buk-gu Gwangju 61186 Republic of Korea; f Department of System Semiconductor Engineering, Sangmyung University 31 Sangmyeongdae-gil, Dongnam-gu Cheonan Chungcheongnam-do 31066 Republic of Korea; g Department of Applied Chemistry, University of Seoul Seoul 02504 Republic of Korea

## Abstract

Near-infrared colloidal nanocrystals (NIR-CNCs) have been widely utilized in optoelectronic applications due to their exceptional optical properties and suitability for mass production. However, their practical application is often hindered by poor chemical stability and suboptimal electronic properties. In this work, four different surface ligand systems—insulating ligands, organic molecular linkers, inorganic molecular linkers, and matrix-type ligands—were systematically investigated to evaluate their effects on the transport and recombination behavior of NIR-CNCs *via* photoinduced carriers. While molecular linkers enhance transport behavior by improving electronic coupling, they tend to induce photoinduced charge carrier accumulation under AM1.5 illumination due to a high degree of Fermi-level pinning caused by unfavorable electronic structures. In contrast, the matrix-type band-like transport ligand significantly reduced dark current and hysteresis characteristics in CNCs, demonstrating superior performance. Impedance and capacitance analyses revealed that the matrix-type ligand, with its multiple carrier separation pathways, enhanced carrier transport through sub-states facilitated by amorphous MoS_*x*_ and effectively passivated CNC trap states, thereby reducing the Fermi-level pinning effect. This approach dramatically suppressed hot carrier-induced trap state generation, minimized photoinduced recombination, and improved operational stability. Overall, this study presents a significant advancement in developing cost-efficient, chemically stable NIR optoelectronic devices with outstanding electronic properties.

## Abstract 2

The new matrix-type ligand system enhances charge carrier transport between particles and passivates defects on particle surfaces by establishing a band-like state.

## Graphical abstract

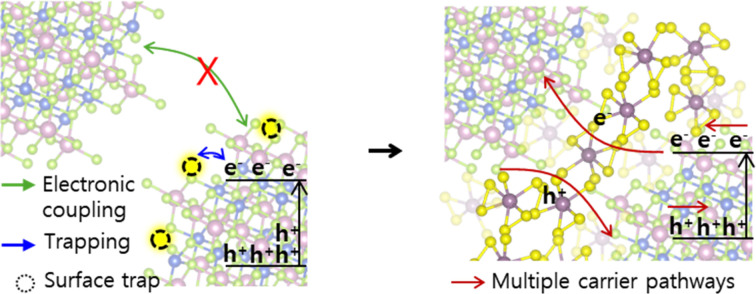

## Introduction

Near-infrared colloidal nanocrystals (NIR-CNCs) have attracted considerable attention due to their flexibility in device applications, cost-effectiveness, scalability for mass production, ease of control over electrical and optical properties, and adaptability for integration into various matrices.^1,2^ These characteristics make NIR-CNCs suitable for a wide range of applications, including thermal imaging for medical diagnostics,^3^ forward-facing sensors in autonomous vehicles, night vision in military contexts, motion detection sensors, and solar cells.^4^ Lead chalcogenides (PbX, where X = S, Se, or Te) and cadmium chalcogenides (CdX, where X = S, Se, or Te) are commonly employed as NIR-CNCs due to their narrow bulk band gaps, large excitonic Bohr radii, and capability for multiple exciton generation.^5–8^ However, their practical use is restricted by environmental compatibility concerns (*e.g.*, toxicity and safety) and stability, particularly in core-type CNCs.

Recently, copper indium chalcogenides (CuInX_2_, X = S, Se, or Te) have emerged as promising candidates for NIR-CNCs, offering high absorption coefficients (∼10^5^ cm^−1^), enhanced environmental compatibility, a direct band gap extending from the visible to the near-infrared range, and excellent tunability.^9^ Additionally, CuInSe_2_ (CISe_2_) CNCs exhibit remarkable structural tolerance, which confers improved sustainability even when stoichiometric mismatches or trap state formation occur, setting them apart from other core-type CNCs.^10^ As all NIR-CNCs require a ligand exchange process due to the insulating nature of long aliphatic ligands used during synthesis, the structural robustness of CISe_2_ broadens the range of feasible ligand exchange methods, including those applicable under harsh conditions. This robustness also enhances the controllability of surface carrier separation pathway of CISe_2_ CNCs through post-treatment.

On the other hand, the low defect formation energy of CISe_2_ leads to the generation of a significant number of surface defects. Both shallow and deep trap states can easily form, depending on the surface state of CISe_2_ CNCs.^11^ This feature hinders the performance of CISe_2_ in NIR applications, resulting in high dark leakage current and severe carrier recombination, which negatively impact the detectivity of photodetectors. Previous studies have demonstrated that a core–shell structure (*e.g.*, CuInS_2_/ZnS) can effectively reduce the number of surface defects in CISe_2_ CNCs, thereby improving photodetector performance. Embedding these core–shell CNCs in an organic matrix further enhances both photoresponsivity and detectivity.^12^ However, the limited range of shell types, specifically those with type-I core–shell band structures, restricts further advancements in the light-detection capabilities of CISe_2_ CNCs. This limitation arises from carrier transport occurring *via* tunneling, which may hinder effective carrier separation from adjacent layers.

Other studies focus on utilizing ligand exchange with organic and inorganic ligands, both of which effectively reduce the interparticle distance of CNCs. This reduction induces efficient electronic coupling between CNCs, improving carrier transport behavior and enhancing the conductivity of the resulting films. Specifically, inorganic ligands, such as metal chalcogenide complexes, bind effectively to the CNC surface, providing electrostatic stabilization. The minimized interparticle distance facilitates strong coupling between CNCs, enabling faster carrier separation.^13^ However, relying solely on electronic coupling between CNCs as a carrier separation pathway is insufficient to overcome performance bottlenecks in CISe_2_ CNC-based photodetectors, particularly due to hot carrier generation in NIR CNCs. When external bias or light intensity is increased, the concentration of photocarrier rises and becomes localized at the surface due to limited available states. This accumulation of carriers at the surface leads to recombination processes, despite the strong electronic coupling between CNCs. This phenomenon is indirectly observed as a loss of mobility in the film state. Even after annealing, the mobilities in NIR CNC films are still much lower than those of bulk materials, mainly due to hot carrier induced coulomb scattering.^14^ Therefore, it is important not only to adjust the distance between particles but also to improve surface states to prevent carrier accumulation at the edges of CISe_2_ CNCs and ensure effective charge carrier transfer in these films.

Since the optimal type of ligand for efficient charge carrier separation and reduced surface recombination remains unclear, this study applied four different types of surface ligands to CISe_2_ CNC-based photodetectors: oleyl amine (OL) for bare CNCs, 3-mercaptopropionic acid (MPA) as an organic molecular linker, tetrathiomolybdate (MoS_4_^2−^) as an inorganic molecular linker, and amorphous molybdenum sulfide (a-MoS*x*) as a matrix-type linker. The photo-detectivity of the lateral-type photodetectors was demonstrated as 1.02 × 10^5^ cm Hz^1/2^ W^−1^ for OL_CNCs, 7.73 × 10^5^ cm Hz^1/2^ W^−1^ for MPA_CNCs, 1.95 × 10^5^ cm Hz^1/2^ W^−1^ for MoS_4_^2−^_CNCs, and 6.28 × 10^6^ cm Hz^1/2^ W^−1^ for a-MoS*x*/CNCs, respectively. The devices incorporating a-MoS_*x*_/CNCs materials demonstrated the highest detectivity with 0.146 A W^−1^ responsivity, coupled with significantly enhanced response times, including accelerated rise and decay times, compared to their molecule-linked CNC counterparts. To elucidate the mechanisms responsible for the superior photodetection performance of a-MoS_*x*_/CNCs materials, the carrier separation pathways in each system were systematically investigated. Capacitance analysis under both illuminated and dark conditions revealed substantial photo-induced surface recombination in molecule-linked CNCs. In contrast, a-MoS_*x*_/CNCs film exhibited negligible increases in surface recombination, highlighting their exceptional charge carrier management under illumination, which is directly associated with enhanced photodetection performance.

## Result and discussion

**Fig. 1:**
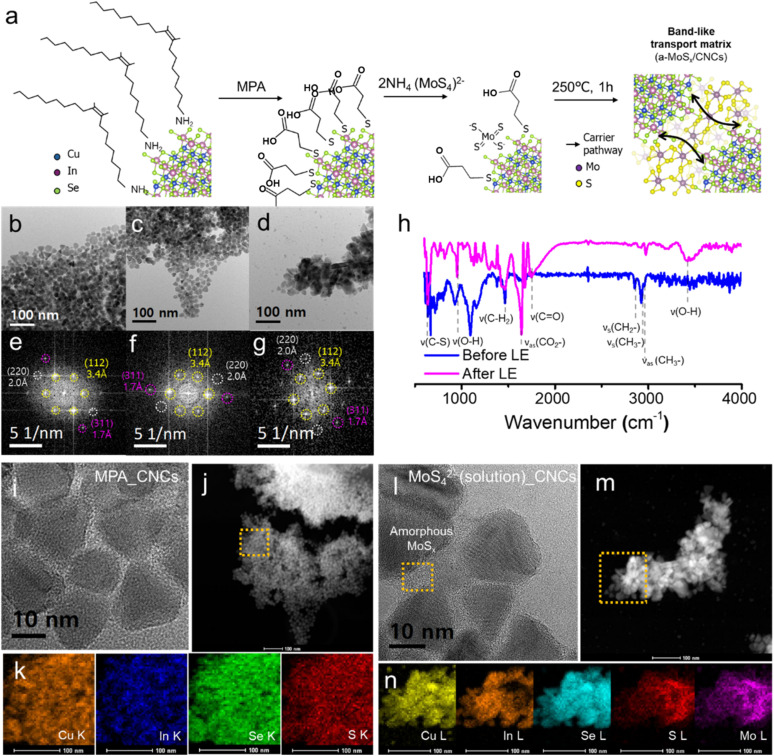
(a) A schematic illustration of preparation of a-MoS_*x*_/CNCs *via* two-step ligand exchange reaction. The TEM images of (b) OL_CNCs, (c) MPA_CNCs, and (d) MoS_4_^2−^(solution)_CNCs, with FFT signals of (e) OL-CNCs, (f) MPA_CNCs and (g) MoS_4_^2−^(solution)_CNCs. (h) FT-IR spectrum of OL_CNCs (before LE, pink solid line) and a-MoS_*x*_/CNCs (after LE, blue solid line). (i) HR-TEM image and (j) TEM dark-field image of MPA_CNCs with corresponding (k) EDX color mapping image. (l) HR-TEM image and (m) TEM dark-field image MoS_4_^2−^(solution)_CNCs with corresponding (n) EDX color mapping image.

Fig. 1a illustrates the schematic representation of the ligand exchange method used to produce a-MoS_*x*_/CNCs materials. OL_CNCs, MPA_CNCs, and MoS_4_^2−^_CNCs were fabricated using previously reported methods,^15^ as detailed in the experimental section. Each system was carefully selected to investigate the carrier separation and surface recombination behavior under AM1.5 illumination and their corresponding impact on photodetection performance. MPA_CNCs was chosen as an organic molecular linker to establish moderate electronic coupling between CNCs, while MoS_4_^2−^_CNCs was selected as an inorganic molecular linker to provide stronger electronic coupling. On the other hand, the matrix-type ligand system, a-MoS_*x*_/CNCs, was designed to study carrier separation and surface recombination behaviors, facilitated by band-like transport. As the direct counterpart to the matrix-type ligand system, MoS_4_^2−^_CNCs enables the investigation of photoinduced charge carrier behavior and its dependence on the separation pathway. The a-MoS_*x*_/CNCs film was synthesized through a two-step ligand exchange process involving MPA and MoS_4_^2−^. This approach utilized the well-reported transformation of the MoS_4_^2−^ ligand into amorphous MoS_*x*_ upon annealing at temperatures exceeding 160 °C,^16^ with its transformation behavior precisely controlled by adjusting the thermal annealing conditions. Initially, a solution-phase ligand exchange process with MPA was performed on the as-synthesized CNCs, followed by film fabrication *via* the spin-coating method. To create a multi-separation pathway for photocarriers, an additional solid-phase ligand exchange process was carried out using a MoS_4_^2−^ solution to establish band-like transport between the CNCs and a-MoS_*x*_. The layer-by-layer deposition was repeated five times to fabricate the a-MoS_*x*_/CNCs film. Finally, the film was annealed at 250 °C under an inert atmosphere, a condition specifically chosen to fabricate an ultrathin amorphous MoS_*x*_ matrix with minimal defects. The CNCs with various ligand exchange process were examined using high-resolution transmission electron microscopy (HRTEM) images, as shown in Fig. 1b–d, to compare the size, shape, and crystal structure of CISe_2_ CNCs after treatment. The size variation of as-synthesized CNCs ranged from 10 to 20 nm, consistent with previous reports. Fourier transform (FFT) signals, (112), (220) and (311) facets were observed with a lattice distance of 3.4 Å, 2.0 Å and 1.7 Å coinciding with the CISe_2_ chalcopyrite structure (Fig. 1e–g).^17^ After the ligand exchange process, no significant changes were observed in the size or crystal structure of CISe_2_ for both MPA_CNCs (Fig. 1c and f) and a-MoS_*x*_/CNCs (Fig. 1d and g), suggesting that the size effect and phase-driven physical properties are negligible relative to the type of ligand exchange reaction.

The successful ligand exchange for a-MoS_*x*_/CNCs was confirmed by Fourier-transform infrared spectroscopy (FT-IR), as shown in Fig. 1h. Prior to the ligand exchange reaction, two distinguishable peaks were observed at 1463 cm^−1^ and 2780–3000 cm^−1^, respectively. The peak at 1463 cm^−1^ corresponded to the –CH_2_ bending vibration, while the peaks in the 2854 cm^−1^, 2924 cm^−1^, and 2956 cm^−1^ were attributed to the –CH_2_ symmetric, –CH_3_ symmetric, and –CH_3_ asymmetric stretching vibration which are characteristic of the oleyl group in aliphatic ligands.^18^ Following the two-step ligand exchange process, the intensities of these peaks significantly decreased, indicating a reduction in oleyl amine on the surface of CISe_2_. Moreover, new peaks emerged at 3400 cm^−1^, 1753 cm^−1^, 1641 cm^−1^, 948 cm^−1^, and a prominent peak at 638 cm^−1^, corresponding to the H-bonded OH stretch, C=O stretch, asymmetric CO_2_^−^ stretch, out-of-plane bending mode of O–H, and C–S bonding, respectively.^19^ Notably, no absorption was observed in the 2550 cm^−1^ region, corresponding to S–H bonding, indicating the cleavage of S–H bonds during the ligand exchange process. This suggests the predominance of metal–sulfur bonds over metal-COOH groups in the system.^20^ These results confirm the successful replacement of oleyl amine ligands with MPA ligands, which remained intact during the secondary ligand exchange process with MoS_4_^2−^. The HRTEM (Fig. 1i and j) and associated energy-dispersive X-ray spectroscopy (EDX) mapping were further conducted to confirm the ligand exchange behavior of organic and inorganic linker. EDX elemental mapping of S from MPA demonstrated a homogeneous distribution after the MPA ligand exchange, like that of Cu, In, and Se, which are components of CNCs (Fig. 1k). On the other hand, the solution-phase MoS_4_^2−^ ligand exchange process revealed the presence of amorphous MoS_*x*_-matrix between the CNCs, unlike MPA_CNCs, which formed from MoS_4_^2−^ activated by electron beam irradiation (Fig. 11n). This observation confirms the successful amorphization of MoS_4_^2−^ using our method. Interestingly, the solution-phase MoS_4_^2−^ exchange exhibited higher intensities of Mo and S near the aggregated CNCs, unlike the MPA treatment, while EDX mapping of In revealed a decrease in intensity near the aggregated CNCs. This suggests that the solution-phase MoS_4_^2−^ treatment is not uniformly deposited on the surface of the CNCs. Negligible peak shift and broadening in the X-ray photoelectron spectroscopy (XPS) spectrum of CNCs treated with solution-phase MoS_4_^2−^ confirm the absence of a significant increase in surface defects during the ligand exchange process (Fig. S1†), consistent with the EDX observations. The solution-phase process causes excess MoS_4_^2−^ to accumulate near aggregated CNCs, resulting in inhomogeneous film formation. Therefore, we optimized the two-step ligand exchange method by combining MPA treatment with a solid-state ligand exchange for MoS_4_^2−^, rather than using the solution-phase method. This approach proved more effective in fabricating homogeneous photodetectors.

**Fig. 2:**
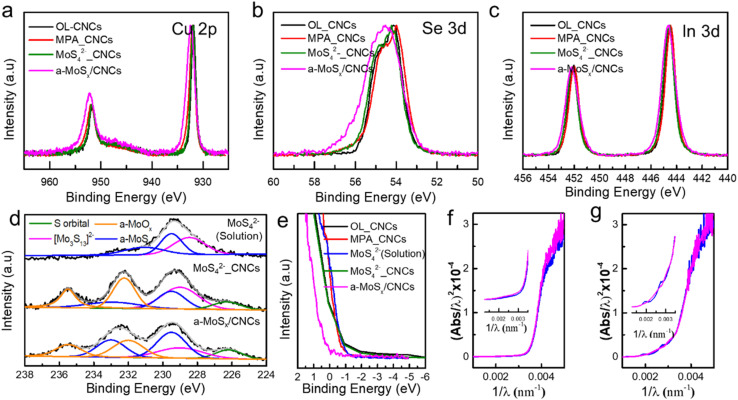
XPS core-level spectrum of (a) Cu 2p, (b) Se 3d and (c) In 3d of OL-CNCs, MPA_CNCs, MoS_4_^2−^_CNCs and a-MoS_*x*_/CNCs respectively. (d) Mo 3d core-level spectrum of MoS_4_^2−^(solution)_CNCs, MoS_4_^2−^_CNCs and a-MoS_*x*_/CNCs. (e) Valence band spectrum of OL-CNCs, MPA_CNCs, MoS_4_^2−^(solution)_CNCs, MoS_4_^2−^_CNCs and a-MoS_*x*_/CNCs. DASF spectrum of (f) MPA_CNCs and (g) a-MoS_*x*_/CNCs.

XPS analysis was performed on all samples, including OL_CNCs, MPA_CNCs, MoS_4_^2−^_CNCs, and a-MoS_*x*_/CNCs, after annealing at 250 °C to assess defect formation in each system. Since CISe_2_ CNCs tend to form off-stoichiometric compositions, which are known to generate defects due to their low defect formation energy, the defect generation associated with each ligand exchange method was examined. These defects (*e.g.*, type, concentration) are expected to significantly influence charge transport behavior, which is a key parameter for altering photodetector performance.^13^ In the core-level spectra of Cu 2p for OL_CNCs, MPA_CNCs, and MoS_4_^2−^-CNCs, two main peaks at 951.7 eV and 931.8 eV, corresponding to Cu 2p_1/2_ and Cu 2p_3/2_, respectively, were observed (Fig. 2a). These three CISe_2_ CNCs exhibited a slight red-shift compared to stoichiometrically accurate CISe_2_ from previous studies,^21^ indicating the Cu-deficient nature of our synthesized materials and the presence of defect pairs, (2V_Cu_^−^ and In_Cu_^2+^), which have the lowest defect formation energy.^10,11^ Consequently, the molecular linkers (MPA and MoS_4_^2−^) did not induce notable charge redistribution on the Cu 2p orbital, as both MPA_CNCs and MoS_4_^2−^_CNCs showed no significant binding energy shifts in the Cu 2p core-level spectra when compared to OL_CNCs (CISe_2_ without ligand exchange processing). After the two-step ligand exchange process (*i.e.*, a-MoS_*x*_/CNCs), a blue shift in the binding energy of Cu 2p orbital was observed, along with an increase in full width half maximum (FWHM). The peak positions were similar to those of stoichiometrically accurate CISe_2_, but exhibited greater peak broadening compared to CISe_2_. The peak broadening in a-MoS_*x*_/CNCs is attributed to band bending, which results from the band-like transport characteristics of the matrix-type a-MoS_*x*_. Thus, the matrix-type ligand is expected to reduce surface defects and promote charge redistribution of Cu–Se, influenced by the adjacent S-enriched environment, thereby improving the electronic properties of the material. A similar trend was observed in the Se 3d (Fig. 2b) and In 3d (Fig. 2c) orbitals. The core-level spectra of Se 3d and In 3d exhibited shifts to higher binding energies and increased FWHM in a-MoS_*x*_/CNCs compared to the other samples.^22^ It is noteworthy that the shift in binding energy for In 3d appeared smaller compared to Cu 2p and Se 3d, as the In–Se bond exhibits weaker covalency than the Cu–Se bond.^21^

To elucidate the role of the a-MoS_*x*_ matrix within CNC thin films, the Mo 3d core-level spectra of all MoS_4_^2−^-treated CNCs were investigated after annealed at 250 °C under inert conditions. This included MoS_4_^2−^ treated in solution phase (MoS_4_^2−^(solution)_CNCs), MoS_4_^2−^ treated in solid phase (MoS_4_^2−^_CNCs), and MPA-MoS_4_^2−^ two-step ligand treated CNCs (a-MoS_*x*_/CNCs). Ammonium tetrathiomolybdate transforms into various compositions, including crystalline MoS_2_ (trigonal or hexagonal), crystalline S-deficient MoS_*x*_ (*x* < 2), and S-rich MoS_*x*_ amorphous phases, depending on the thermal annealing conditions.^22,23^ Investigating the chemical bonding and oxidation states of molybdenum is crucial, as the electronic properties of these Mo–S derivatives depend heavily on their compositions.^25^ MoS_4_^2−^(solution)_CNCs, which was treated with solution phase MoS_4_^2−^, exhibited dominant peaks at 229.4 eV and 232.4 eV, confirming the formation of the [Mo_3_S_13_]^2−^ cluster with a minor amount of amorphous a-MoS_*x*_ rather than the 2H-MoS_2_ phase (Fig. 2d).^24,25^ This is consistent with previous studies, which indicate that MoS_4_^2−^ initially forms MoS_3_ and transitions to the 2H-MoS_2_ phase only at temperatures exceeding 500 °C. The significant intensity difference between the Mo 3d_5/2_ and Mo 3d_3/2_ peaks further confirms the molecular nature of MoS_*x*_ in MoS_4_^2−^(solution)_CNCs, as opposed to a crystalline structure. For MoS_4_^2−^_CNCs, which was treated with MoS_4_^2−^ in solid phase, dominant peaks at 229.4 eV and 232.4 eV were observed, indicating a high concentration of [Mo_3_S_13_]^2−^ clusters, along with a slightly increased proportion of a-MoS_*x*_. Additionally, partial amorphous oxide peaks at 232.4 eV and 235.4 eV were also detected, resulting from oxygen trapped during the spin-coating process that oxidized molybdenum during annealing. Notably, the Mo 3d spectra of a-MoS_*x*_/CNCs exhibited blue-shifted peaks at 229.6 eV and 232.7 eV, characteristic of an amorphous, sulfur rich MoS_*x*_ phase.^26^ This increased portion of a-MoS_*x*_ is attributed to the homogeneous deposition of MoS_4_^2−^, which promotes the formation of matrix-type a-MoS_*x*_. Similarly, the S 2p orbital displayed a blue-shifted peak in a-MoS_*x*_/CNCs, which is attributed to an increased fraction of S–S bridges compared to S–S terminals (Fig. S2†).^24^ This shift provides evidence for the predominance of an amorphous phase in a-MoS_*x*_/CNCs. Furthermore, the reduced intensity of the amorphous oxide peaks at 232.4 eV and 235.4 eV indicates enhanced material stability.

This improved stability suggests that the MPA ligands on CNCs facilitate the uniform anchoring of MoS_4_^2−^ on their surfaces, resulting in an enriched MoS_4_^2−^ environment. The hydrogen bonding provided by the COOH groups, combined with the increased London dispersion forces from the benzene rings of MPA, prevents CNC aggregation while providing sufficient space for the formation of Mo–S derivative networks.^27^ Consequently, the enriched MoS_4_^2−^ environment effectively occupies Se vacancies or incorporates into voids and grain boundaries. Upon annealing, this enriched MoS_4_^2−^ undergoes a successful transformation into an amorphous MoS_*x*_ matrix, which can facilitate band-like transport. This observation was further supported by the valence band spectra shown in Fig. 2e. The Fermi level of a-MoS_*x*_/CNCs shifted by approximately 0.8 eV compared to that of other samples, which is attributed to the reduction of defect states induced by a-MoS_*x*_ matrix. In contrast, MPA_CNCs and MoS_4_^2−^_CNCs exhibited no significant change near the Fermi level relative to OL_CNCs. The defect states in CISe_2_, which exist within the NIR band gap, are located above the valence band and beneath the conduction band. These defect states tend to neutralize the charge and compensate each other, leading to Fermi level pinning, which significantly affects photocarrier transport behavior.^28^ Due to strong Fermi level pinning, the band edge state and band tail remained unchanged for MPA_CNCs or MoS_4_^2−^_CNCs (Fig. 2f). However, a-MoS_*x*_/CNCs exhibited blue shift (Fig. 2g), indicating the removal of existing vacancies and strong coupling between the Se core and MoS_*x*_, attributed to a decrease in the electron cloud density of Se.^29^ These findings indicate that the two-step ligand exchange method is highly effective in fabricating a matrix-type band-like ligand system. This system enhances charge carrier transport efficiency by physically and electronically passivating defects, thereby significantly improving photodetection performance.

**Fig. 3:**
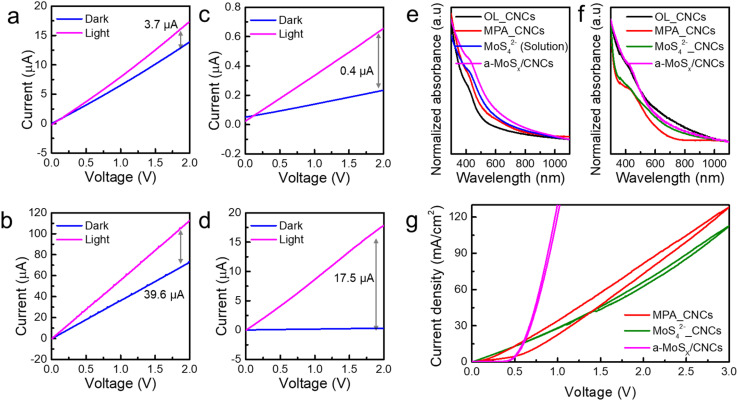
Static-state responsivity for (a) OL_CNCs, (b) MPA_CNCs, (c) MoS_4_^2−^(solution)_CNCs and (d) a-MoS_*x*_/CNCs using a Xenon lamp at 100 mW cm^−2^ intensity. (e) The normalized absorption spectrum measured in solution phase OL_CNCs (black), MPA_CNCs (red), MoS_4_^2−^(solution)_CNCs (blue) and a-MoS_*x*_/CNCs (pink). (f) The normalized absorption spectrum measured in thin-film phase OL_CNCs (black), MPA_CNCs (red), MoS_4_^2−^_CNCs (green), and a-MoS_*x*_/CNCs (pink). (g) The hysteresis curve of MPA_CNCs (red), MoS_4_^2−^_CNCs (green) and a-MoS_*x*_/CNCs (pink).

Finally, photoconductors based on four different types of CNC networks were fabricated to evaluate their photo-induced current. Scanning electron microscope (SEM) images were obtained to examine the uniformity of the surface morphology of the photodetector (Fig. S3†). As no significant differences were observed in factors that could influence device characteristics, such as grain boundaries or surface roughness, the measured photodetector performance can be primarily attributed to the ligand systems within the active layer. A lateral-type channel was employed as shown in Fig. S4† for this study, as opposed to layered-structured devices, to analyze charge separation and photoinduced surface recombination behavior within the CNC networks, depending on the surface ligand system. Photocurrent measurements were conducted under light irradiation using a Xenon lamp at 100 mW cm^−2^ intensity (AM1.5), as shown in Fig. 3a–d. To prevent the overestimation of photocurrent in the photodetector, the device was exposed to light over an area larger than its active area.^30,31^ For OL_CNCs, MPA_CNCs, MoS_4_^2−^(solution), and a-MoS_*x*_/CNCs-based photodetectors, measured at a 2V bias and obtained by subtracting the dark current, exhibited the following trend: MPA_CNCs (39.6 μA) > a-MoS_*x*_/CNCs (17.5 μA) > OL_CNCs (3.7 μA) > MoS_4_^2−^(solution) (0.4 μA). In contrast, the dark current followed a different trend: MPA_CNCs (73.0 μA) > OL_CNCs (13.5 μA) > a-MoS_*x*_/CNCs (0.33 μA) > MoS_4_^2−^(solution) (0.23 μA). Notably, MPA_CNCs demonstrated the highest photocurrent and dark current due to the increment of acceptor states, therefore both photo-generated and majority charge carrier increased. The dark current in NIR materials is closely related to the number of majority charge carriers. As the trap density increases in NIR-CNCs, these trap states behave as dopants, contributing to a higher concentration of majority charge carriers. The presence of these trap states facilitates the generation of thermally excited carriers and promotes interparticle band to band transition between neighboring CNCs, ultimately leading to an increase in the dark current.^32^ Consequently, a 10-order magnitude increase in both photo and dark currents was observed in MPA_CNCs compared to OL_CNCs, which can be attributed to the synergistic effect between MPA doping and intrinsic p-type defects. On the other hand, MoS_4_^2−^(solution) (Fig. 3c) and MoS_4_^2−^_CNCs (Fig. S5†) demonstrated decreases in both photo (0.92 μA) and dark currents (1.64 μA). The morphological inhomogeneity observed in the TEM images negatively impacted the photodetection behavior of the MoS_4_^2−^(solution) film, and MoS_4_^2−^_CNCs (solid state exchange) exhibited reduced electronic coupling between CNCs, attributed to the presence of a large amount of residual OL. Interestingly, a-MoS_*x*_/CNCs demonstrated significantly suppressed dark current due to effective defect passivation, ensuring stability throughout operation (Fig. S6a†). Although MPA_CNCs exhibited the highest photoresponsivity, their dark current gradually increased during operation (Fig. S6b†). This increase was attributed to hot carriers further enhancing trap states, leading to universal mobility degradation.^33^ Once the dark current of MPA_CNCs photodetector reached approximately the ∼1 mA scale, the device ceased to exhibit any photoresponsivity. The robustness of a-MoS_*x*_/CNCs allowed its photodetector to maintain consistent operation even after one month of storage (Fig. S7a†) and 100 operational cycles (Fig. S7b†).

To clarify the reasons behind the enhanced photodetection behavior and robustness of a-MoS_*x*_/CNCs, the optical band gap of each system was measured and analyzed. The normalized absorption spectrum of CISe_2_ with various ligands is depicted in Fig. 3e. The absorption shoulder peak at 460 nm corresponding to the excitation peak of CISe_2_, was preserved across all samples.^21^ This suggests that the long-range electronic structure and structural properties of CNCs were maintained during the three different ligand exchange treatments, consistent with the results observed in TEM investigations. The absorption spectra of all samples showed that absorption near the 460 nm was still preserved even after annealing, which is distinguishable features of CISe_2_ (Fig. 3f). Therefore, the enhanced photodetection properties of a-MoS_*x*_/CNCs were not attributed to the change of intrinsic band structure which is well coincident with ligand chemistry study. To investigate the effect of defect presence, hysteresis curve of each sample was obtained as shown in Fig. 3g. MPA_CNCs exhibited the largest hysteresis, suggesting that severe number of trap states, which influences the material's charge transport properties.^34^ Low conductivity with low degree of hysteresis of MoS_4_^2−^_CNCs can be attributed to the synergistic effect of interparticle distance modulation and the additional donor states introduced by MoS_4_^2−^ derivative (*i.e.*, [Mo_3_S_13_]^2−^ and a small portion of MoS_*x*_). This combination enhances charge separation, which explains the smaller hysteresis observed in MoS_4_^2−^_CNCs compared to MPA_CNCs. On the other hand, a-MoS_*x*_/CNCs exhibiting the smallest hysteresis which indicates the presence of fewer trap states, suggesting that the a-MoS_*x*_ matrix maintains relatively stable electronic properties. With lower defect density and optimized electronic structure, the a-MoS_*x*_/CNCs demonstrate improved charge transport and material stability, making them a promising candidate for enhanced performance in photodetector applications.

Since amount of a-MoO_*x*_ slightly different between MoS_4_^2−^_CNCs and a-MoS_*x*_/CNCs, we further investigated photodetection behavior of a-MoS_*x*_/CNCs samples under two different annealing environments: a-MoO_*x*_ fabricated under air condition (a-MoS_*x*_/CNCs_Air), and a-MoO_*x*_ fabricated with MeOH rich film under Ar condition (a-MoS_*x*_/CNCs_MeOH_Ar). As shown in Fig. S8a,† the sample annealed in air exhibited a significantly stronger a-MoO_*x*_ peak compared to a-MoS_*x*_/CNCs annealed under Ar, both with and without residual MeOH. The a-MoS_*x*_/CNCs_Air exhibited the highest MoO_*x*_ intensity and the lowest S orbital intensity. Since the oxygen source originated externally, a-MoS_*x*_/CNCs_Air primarily formed surface a-MoO_*x*_ during the annealing process. In contrast, a-MoS_*x*_/CNCs_MeOH_Ar displayed lower a-MoO_*x*_ intensity and higher S orbital intensity compared to a-MoS_*x*_/CNCs_Air. However, when compared to a-MoS_*x*_/CNCs_Ar, a-MoS_*x*_/CNCs_MeOH_Ar showed increased a-MoO_*x*_ intensity and decreased S orbital intensity, indicating reduced S–S bonding. This suggests that most of the oxidation in a-MoS_*x*_/CNCs_MeOH_Ar originated from residual MeOH trapped within the film, leading to the formation of a-MoO_*x*_ within the CNCs network and disrupting the continuity of the MoS_*x*_ matrix. The photodetection performance of a-MoS_*x*_/CNCs_Air and a-MoS_*x*_/CNCs_MeOH_Ar samples clearly demonstrated the distinct effects of surface a-MoO_*x*_ and network-embedded a-MoO_*x*_ within the CNCs network (Fig. S8b and c†). The a-MoS_*x*_/CNCs_Air sample exhibited a slight reduction in photocurrent and an increase in dark current, whereas a-MoS_*x*_/CNCs_MeOH_Ar showed a significantly reduced photocurrent with negligible changes in dark current compared to a-MoS_*x*_/CNCs_Ar. These results suggest that surface a-MoO_*x*_ has minimal impact on the carrier transport behavior of CISe_2_ CNCs, while a-MoO_*x*_ embedded within the network severely disrupts charge transport. Based on these findings, the differences in photodetection behavior between MoS_4_^2−^_CNCs and a-MoS_*x*_/CNCs are primarily attributed to the disruption of the a-MoS_*x*_ matrix caused by embedded a-MoO_*x*_.

**Fig. 4:**
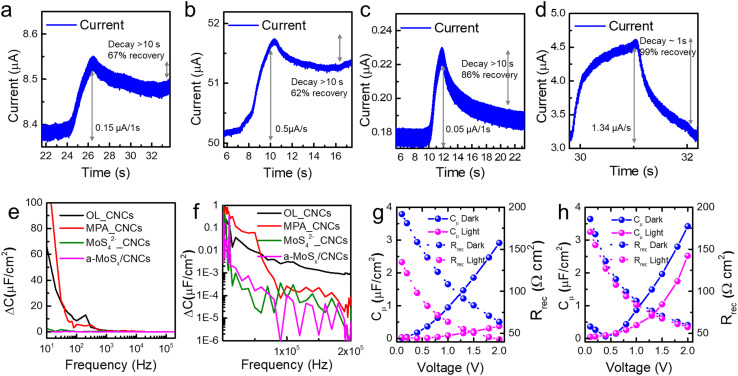
On–off response of photodetectors with (a) OL_CNCs, (b) MPA_CNCs, (c) MoS_4_^2−^_CNCs (solution), and (d) a-MoS_*x*_/CNCs. Photoinduced capacitance as a function of frequency for CNCs with four different ligand systems presented on (e) a linear scale and (f) a logarithmic scale: OL_CNCs, MPA_CNCs, MoS_4_^2−^_CNCs, and a-MoS_*x*_/CNCs. Voltage *versus* chemical capacitance and recombination resistance plots under light and dark conditions for (g) MPA_CNCs and (h) a-MoS_*x*_/CNCs.

Interestingly, the increased trap states lead to surface recombination loss under dark conditions, while enhanced recombination for photoinduced carriers under AM1.5 illumination. The on–off behavior of each sample clearly demonstrated distinct charge transport dynamics. The static-state responsivity for each sample was determined as follows (Fig. 3a–d): 1.8 μA (OL_CNCs), 13.5 μA (MPA_CNCs), 0.2 μA (MoS_4_^2−^_CNCs), and 8.8 μA (a-MoS_*x*_/CNCs). Under dynamic-state conditions, the responsivity followed a trend of 0.03 μA for OL_CNCs, 0.33 μA for MPA_CNCs, 0.05 μA for MoS_4_^2−^_CNCs, and 1.34 μA for a-MoS_*x*_/CNCs (Fig. 4a–d). Remarkably, the responsivity of the a-MoS_*x*_/CNCs-based photodetector was quite similar with that of MPA_CNCs during dynamic-state measurements. In addition, OL_CNCs and MPA_CNCs exhibited dramatically elevated off-current, while MoS_4_^2−^_CNCs and a-MoS_*x*_/CNCs showed stable on-to-off behavior during four cycles, indicating more efficient charge separation and retention (Fig. S9†). The rise and decay times were significantly longer for OL_CNCs and MPA_CNCs compared to MoS_4_^2−^_CNCs (which showed an 86% recovery at 10 s but did not fully decay) and a-MoS_*x*_/CNCs (with a decay time of approximately 1 second). These results suggest that the MoS_4_^2−^-derived surface modification influences the reduced photoinduced carrier recombination, potentially improving the stability of CNCs and photoinduced charge separation of MoS_4_^2−^_CNCs and a-MoS_*x*_/CNCs compared to OL_CNCs and MPA_CNCs. Additionally, impedance analysis was conducted using an FTO/NiO_*x*_/active materials/GaIn device structure to investigate the photoinduced carrier recombination behavior across different ligand systems. Capacitance changes with frequency were measured under both light and dark conditions (Fig. 4e and f). For MPA_CNCs, a significant capacitance loss was observed primarily in the low-frequency region, suggesting that photoinduced carriers tend to accumulate due to surface recombination processes.^35^ This behavior indicates that acceptor states play a significant role in hindering efficient charge transport in MPA_CNCs by promoting carrier recombination at the surface, which was reported in CISe_2_ passivated with excess sulfur.^36^ In the case of OL_CNCs, a larger capacitance loss was also observed in the low-frequency region. However, the frequency dependence was drastically reduced compared to MPA_CNCs. This behavior suggests that the photoinduced carrier accumulation in OL_CNCs primarily stems from the insulating nature of the oleyl amine ligands. These ligands contribute to surface recombination and influence the morphology-dependent resistance rather than being directly related to trap states (*i.e.*, vacancy and dopant state). On the other hand, for MoS_4_^2−^_CNCs and a-MoS_*x*_/CNCs, the capacitance changes were more evenly distributed across both low and high-frequency regions. Although the capacitance changes were still more pronounced in the low-frequency region, the extent of the changes was significantly smaller than those observed for MPA_CNCs. Additionally, the capacitance differences with and without light for MoS_4_^2−^_CNCs and a-MoS_*x*_/CNCs remained below several nF at frequencies above 1 kHz. This behavior indicates that MoS_4_^2−^_CNCs and a-MoS_*x*_/CNCs exhibit reduced hot carrier generation.^37^ The strong electronic coupling facilitated by MoS_4_^2−^ ligands and the stable electronic properties of the a-MoS_*x*_ matrix effectively minimize the influence of trap states as well as diversifying charge carrier separation pathways. This charge separation can be explained by the rapid drift of charge carriers in the depletion region. By forming a continuous matrix outside the CNCs, it behaves like a bulk material rather than individual molecules, creating a band-like density of states. This feature, combined with the electron-rich characteristics of a-MoS_*x*_, forms a homogeneous junction with the p-type CNCs. Consequently, a depletion region is established at the interface between the a-MoS_*x*_ matrix and CNCs, contributing to the enhanced performance of the photodetector.^38,39^ Interestingly, a slight increase in photoinduced capacitance at high-frequency regions suggests morphological inhomogeneity in MoS_4_^2−^_CNCs, aligning with prior observations. As a result, a-MoS_*x*_/CNCs demonstrates superior efficiency in charge separation and transport compared to MPA_CNCs and MoS_4_^2−^_CNCs.

Moreover, the capacitance and resistance were examined under varying voltage conditions, both with and without light exposure, in the low-frequency range (around 1 kHz). This frequency range was selected because it is particularly sensitive to the effects of surface recombination and charge transport dynamics. Under illumination, MPA_CNCs displayed significant changes in both chemical capacitance and recombination resistance (Fig. 4f).^40^ The resistance decreased notably in the presence of light, indicating enhanced recombination of mobile charge carriers. This reduction in resistance correlates with decreased charge separation efficiency, emphasizing the detrimental role of surface recombination.^41^ Simultaneously, the capacitance dropped, reflecting a reduction in induced polarization likely caused by diminished separation of photoinduced carriers. These findings demonstrate that the high density of acceptor states in MPA_CNCs results in imbalance of majority and minor carrier, thereby hindering efficient carrier transport under AM1.5 illumination. In contrast, a-MoS_*x*_/CNCs exhibited minimal changes in capacitance and resistance under similar conditions (Fig. 4g). This suggests that photoinduced recombination is effectively suppressed in a-MoS_*x*_/CNCs, leading to more stable charge transport through multiple pathways. The minimal variations in capacitance and resistance highlight the ability of the a-MoS_*x*_ matrix to mitigate trap-state-induced recombination. This improvement can be attributed to strong electronic coupling facilitated by the molecular linker and the band-like transition between CNCs and the amorphous MoS_*x*_ phase, as observed in the XPS analysis. These synergistic effects enhance charge separation and promote efficient photocarrier transport in a-MoS_*x*_/CNCs, making them a robust material for optoelectronic applications. To further evaluate the applicability of this system, additional photodetection performance were conducted under low-energy light exposure. Photodetection and impedance studies across four different ligand systems confirmed that the a-MoS_*x*_ matrix ligand system effectively passivates defects both physically and electronically. This suggests its potential to overcome the performance limitations of NIR photodetectors caused by unintended hot carrier generation from narrow bandgap materials. As shown in Fig. S10,† a-MoS_*x*_/CNCs based photodetector demonstrated a responsivity of 0.150 A W^−1^ and a specific detectivity of 6.63 × 10^6^ cm·Hz^1/2^·W^−1^ under low-energy light illumination (660 nm laser with 200 mW cm^−2^ light intensity). These findings indicate that the matrix-type ligand system are able to enhance NIR photodetector performance by providing a continuous a-MoS_*x*_ matrix that minimizes charge carrier trapping and hot carrier generation under AM1.5 illumination.

In conclusion, this study intensively investigated the distinct electronic coupling behavior and resulting photodetection performance associated with different ligand systems: (1) the original long-chain insulating ligand, OL; (2) a short-chain organic ligand, MPA; (3) a short inorganic molecule with a distinct energy state, the MoS_4_^2−^ ligand; and (4) a combination of short-distance ligands with distinct electronic structures, a-MoS_*x*_/CNCs (MPA + MoS_4_^2−^). OL_CNCs exhibited detrimental effects when applied directly to devices, underscoring a common limitation of traditional colloidal nanomaterial synthesis. To address these shortcomings, short-length ligands, MPA and MoS_4_^2−^, were employed. Although the MPA ligand reduced interparticle distance, it resulted in increased dark current and photoinduced charge accumulation due to carrier imbalance arising from an unfavorable energy level, ultimately degrading photodetection performance. In contrast, MoS_4_^2−^ ligand offered a favorable energy level structure but resulted in severe film non-uniformity due to low colloidal solution stability, leading to an increased interparticle distance and hindered electronic coupling. Consequently, while photoinduced charge accumulation decreased significantly, the responsivity of photodetection also diminished. A solid-state ligand exchange method for the MoS_4_^2−^ ligand was introduced to improve uniformity, but it failed to overcome the limitations imposed by the long chain length of the OL ligand. This suggested that both interparticle distance and appropriate electronic structure should be considered in the selection of ligands. Based on the results, a novel ligand system using a-MoS_*x*_ as a matrix-type linker was developed for CISe_2_ CNC-based photodetectors. This integration achieved the highest detectivity (6.28 × 10^6^ cm·Hz^1/2^·W^−1^) and exhibited stable operation compared to the other systems investigated. The enhanced performance is attributed to the a-MoS_*x*_ matrix's ability to suppress defect-induced hot carrier generation and recombination, originated from effective electronic coupling and band-like carrier transitions facilitated by the amorphous MoS_*x*_ matrix. Capacitance and resistance analyses further corroborated these findings, revealing minimal photoinduced recombination in a-MoS_*x*_/CNCs. These results highlight the transformative potential of a-MoS_*x*_/CNCs in the development of high-performance, durable, and scalable optoelectronic applications.

## Data availability

The data supporting this article have been included as part of the ESI.†

## Author contributions

The manuscript was written through contributions of all authors. H. Kim: conceptualization, formal analysis, writing – original draft, and editing, Y. Jeong: material synthesis and film fabrication, W.-G. Jung, W.-J. Moon: TEM analysis, M. Kim, J. Yang: material characteristic, M. Kim, Y. Han, H. Ko: formal analysis and visualization, S. W. Hwang: methodology, formal analysis, M. J. Kim, J. W. Lee: formal analysis, H. Lee: conceptualization, editing, resources, and supervision. All authors have given their approval to the final version of the manuscript.

## Conflicts of interest

There are no conflicts to declare.

## Supplementary Material

RA-015-D4RA08792E-s001
