# Supplementary material for: Designing multiple charge carrier separation pathways in core-type near infrared colloidal nanocrystal for broadband photodetector
Source: RSC Adv. 2025 Feb 27;15(9):6531–40. doi: 10.1039/d4ra08792e (PMC11865893; doi:10.1039/d4ra08792e)
Supplement: RA-015-D4RA08792E-s001 [file RA-015-D4RA08792E-s001.pdf]

Supplementary Information (SI) for RSC Advances.
This journal is © The Royal Society of Chemistry 2025

Supporting Information

Designing multiple charge carrier separation pathways in core-type near infrared colloidal nanocrystal for broadband photodetector

Hyunjung Kim,[1,2] Yoonji Jeong,[3] Wan-Gil Jung,[5] Minju Kim,[3] Jiyoon Yang,[3] Minseo Kim,[2] Yeonsu Han,[2] Hyun Ko,[4] Sung Won Hwang,[6] Myeong Jin Kim,[7] Jong Woo Lee,[7] Won-Jin Moon[5] and Hanleem Lee[3]*

[1]Research Institute of Basic Sciences, Sungkyunkwan University, 2066 Seobu-Ro, Suwon, 16419, Republic of Korea.

[2]Department of Chemistry, Sungkyunkwan University, 2066 Seobu-Ro, Suwon, 16419, Republic of Korea

[3]Department of Chemistry, Myongji University, 116 Myongji Ro, Yongin, Gyeonggi-do, 17058, South Korea.

[4]Institute for Quantum Biophysics (IQB), Sungkyunkwan University, 2066 Seobu-Ro, Suwon, 16419, Republic of Korea

[5]Korea Basic Science Institute, Gwangju Center, 77 Yongbong-ro, Buk-gu, Gwangju 61186, Republic of Korea

[6]Department of System Semiconductor Engineering, Sangmyung University, 31 Sangmyeongdae-gil, Dongnam-gu, Cheonan 31066, Chungcheongnam-do, Korea.

[7]Department of Applied Chemistry, University of Seoul, Seoul, 02504, Republic of Korea

E-mail: (Hanleem Lee) hanleem@mju.ac.kr

KEYWORDS: $CuInSe_2$, colloidal nanocrystals, photodetector, photocarrier recombination, ligand exchange

Experiment details

Materials. Copper(I) acetylacetonate ($Cu(acac)_2$, 98%, Alfa Aesar), indium chloride ($InCl_3$, 99.9%, Sigma Aldrich), selenium powder - 200 mesh (Se, 99.999%, Alfa Aesar), ammonium tetrathiomolybdate (ATTM, 99.97%, Sigma Aldrich), and tetrabutylammonium hexafluorophosphate ($TBAPF_6$, 98%, Sigma Aldrich) were purchased and stored in a nitrogen-filled glovebox. Oleylamine (OL, 70% grade, Sigma Aldrich) was used after being degassed under vacuum for 1 hour at 110°C. Methanol (MeOH), hexane, toluene, mercaptopropionic acid (MPA, for synthesis, Sigma Aldrich), N-methylformamide (NMF), potassium hydroxide (KOH), acetonitrile (ACN), and isopropyl alcohol (IPA) were used as received.

Synthesis of $CISe_2$ ($CuInSe_2$) CNCs. The synthesis of $CISe_2$ CNCs via the hot-injection method was conducted following a previous study.[1] All reagents were stored and prepared in a nitrogen-filled glovebox. Specifically, 0.2 mmol of $Cu(acac)_2$ (44.2 mg), 0.2 mmol of $InCl_3$ (52.4 mg), and 5 ml of OL were added to a 25 ml three-neck round-bottom flask (RBF). For the selenium pot (Se-pot), 31.6 mg of selenium powder and 8 ml of OL were added to another RBF. After sealing both RBFs with septa, they were transferred out of the glovebox and connected to a Schlenk system. The Se-pot was degassed under vacuum conditions and heated to 110°C for 30 minutes, while the Se-pot was degassed at 80°C. The temperature of the Se-pot was then increased to 250°C under a nitrogen atmosphere, and 5 ml of the metal precursor solution was swiftly injected. Upon injection of the metal precursor solution into the Se-pot, the reaction pot's color changed from clear yellow-orange to dark brown. The heating mantle was then removed to cool the solution down to 100°C. The RBF was rapidly re-equipped with the heating mantle and reheated to 250°C for 1 hour. Finally, the $CISe_2$ solution, which appeared dark brown, was removed from the heating mantle to cool down and terminate the reaction. To purify the as-synthesized $CISe_2$, 5 ml of methanol (MeOH) was added to the crude product, followed by centrifugation at 6000 rpm for 1 minute. Further purification involved rinsing the precipitates with 5 ml of MeOH and 5 ml of hexane, followed by centrifugation at 6000 rpm for 1 minute. Finally, 4 ml of toluene was added to the precipitated $CISe_2$, and the solution was centrifuged at 1000 rpm for 1 minute to obtain homogeneous $CISe_2$ CNCs. The supernatant in toluene was transferred to a vial and stored for further characterization and ligand exchange reactions.

OL_CNCs solution preparation. OL-CNCs were obtained by centrifuging the CISe CNCs solution at 6000 rpm for 1 minute with the addition of 8 ml of MeOH. The precipitates were re-dispersed in 1 ml of toluene to form OL-CNCs ink. For the preparation of OL-CNCs thin films, OL-CNCs ink was dropped onto patterned ITO glass (Ossila) and then spin-coated at 2000 rpm for 50 seconds. Finally, OL-CNCs thin films were obtained by annealing the film at 250°C for 1 hour under nitrogen-filled conditions.

MPA_CNCs solution and thin film preparation. MPA_CNCs were fabricated by modifying a previous method.[2] 1 ml of 0.1 M MPA in methanol solution and 0.1 ml of 0.12 M KOH in methanol solution were added to 4 ml of 0.1 mmol $CISe_2$ in toluene, and the mixture was vigorously stirred. After 1 hour, the solution was transferred into a conical tube, and 2 ml of ethanol was added. The solution was centrifuged at 6000 rpm for 1 minute, and the precipitates were collected and rinsed with a mixture of 50% (v/v) methanol/toluene solution. The precipitates were then dispersed in 1 ml of toluene to form MPA_CNCs ink. For the preparation of MPA_CNCs thin films, MPA_CNCs ink was dropped onto patterned ITO glass (Ossila) and spin-coated at 2000 rpm for 50 seconds. Finally, MPA_CNCs thin films were obtained by annealing the film at 250°C for 1 hour under nitrogen-filled conditions.

MoS_4^{2-}(Solution) solution and thin film preparation. MoS_4^{2-}(Solution) was fabricated by modifying a previous method.[3] A 10 mg/ml solution of ATTM in NMF was added to a solution of 0.1 mmol $CISe_2$ in toluene along with 0.1 ml of 0.12 M KOH in MeOH, and the mixture was vigorously stirred for 1 hour. The MoS_4^{2-}(Solution) samples were then centrifuged at 6000 rpm for 1 minute and rinsed with a mixture of 50% (v/v) hexane/ethanol solution at 6000 rpm for 1 minute. The precipitates were dispersed in 1 ml of IPA to obtain MoS_4^{2-}(Solution) ink. For the preparation of MoS_4^{2-}(Solution)_CNCs thin films, MoS_4^{2-}(Solution)_CNCs ink was dropped onto patterned ITO glass (Ossila) and spin-coated at

2000 rpm for 50 seconds. Finally, MoS_4^{2-}(Solution)_CNCs thin films were obtained by annealing the film at 250°C for 1 hour under nitrogen-filled conditions.

MoS_4_CNCs thin film preparation. MoS_4-CNCs were fabricated by treating ATTM solution onto the OL-CNCs thin film. A 2.5 mg/ml solution of ATTM in MeOH was dropped onto the OL-CNCs thin film and maintained for 2 minutes to allow sufficient exchange of the OL ligand with MoS_4^{2-}. MoS_4^{2-}-CNCs thin film was obtained by spin coating at 2000 rpm for 50 seconds, followed by two additional rinses of residual ligand by dropping MeOH onto the film and then spin coating again. A soft annealing process was conducted at 80°C for 5 minutes, and the above process was repeated 3 times to attain the suitable thickness of MoS_4-CNCs. Finally, MoS_4-CNCs thin film was obtained by annealing the film at 250°C for 1 hour under nitrogen-filled conditions.

a-MoS_x/CNCs preparation. a-MoS_x/CNCs were fabricated by treating ATTM solution onto the MPA-CNCs thin film. A 2.5 mg/ml solution of ATTM in MeOH was dropped onto the MPA-CNCs thin film and maintained for 2 minutes to allow sufficient exchange of the MPA and remained OL ligand with MoS_4^{2-}. a-MoS_x/CNCs thin film was obtained by spin coating at 2000 rpm for 50 seconds, followed by two additional rinses of residual ligand by dropping MeOH onto the film and then spin coating again. A soft annealing process was conducted at 80°C for 5 minutes, and the above process was repeated 3 times to attain the suitable thickness of a-MoS_x/CNCs. Finally, a-MoS_x/CNCs thin film was obtained by annealing the film at 250°C for 1 hour under nitrogen-filled conditions.

Transmission electron microscopy. The TEM sample was prepared by dropping the dilution of 0.1 mmol $CISe_2$ in 4 ml toluene solution multiple times onto a carbon film supported on a 200 mesh copper grid (TED PELLA, INC.), then dried under vacuum overnight. TEM (Tecnai G2 F20 S-Twin, FEI) was used with an acceleration voltage of 200 kV.

Uv-vis spectroscopy and band tail measurement via derivative absorption spectrum fitting method (DASF). For measurement of absorption spectra of CNCs solution, OL_CNCs and MPA_CNCs were dispersed in toluene while MoS_4^{2-}(Solution)_CNCs were dispersed in NMF then measured after equipped in 10mm size of a quartz cuvette. For measurement of absorption spectra of CNCs thin film, OL-CNCs, MPA_CNCs, MoS_4^{2-}(solution), MoS_4_CNCs, and a-MoS_x/CNCs film was fabricated on the slide glass. DASF method was conducted by plotting the

X-ray photoelectron spectroscopy measurement. All the CNCs thin film was faricated on the heavily boron doped silicon wafer with 10x10mm^2 size for measurement of core level and valence band spectra. Thermofisher Scientific Nexsa was used with the 10um~400um spot size, and all the spectra was calibrated with C 1s as 284.8eV,

FT-IR spectroscopy measurement. All the CNCs thin film was fabricated on the heavily boron doped silicon wafer with 10x10mm^2 size and measured by LUMOS II (Bruker)

Photodetector device measurement. For fabrication of photodetector, patterned ITO glass with the 600 μm channel length and 200 μm width was used after washing the substrate with IPA and distilled ionized water. After preparation of CNCs thin film as described above, photodetector devices were equipped in a probe station. Photocurrent of CNCs photodetectors was measured under 1 sun irradiation of xenon arc lamp (100 mW/cm^2). The structure of the as-prepared lateral type photodetector was depicted in Figure S4. Static measurements were conducted using I-V measurements under continuous light exposure. Initially, the dark current was measured over a voltage range of 0 V to 2 V at a scan rate of 10 mV/s. The device was then exposed to 1 sun irradiation for 1 minute, and the photoinduced current was subsequently measured under the same conditions (a voltage range of 0 V to 2 V at a scan rate of 10 mV/s). Dynamic measurements were conducted in constant voltage mode with a chopper applied to the light source. For a-MoS_x/CNC, a cycle of 1-second light exposure followed by 1-second darkness was continuously repeated. For the other samples, a cycle of 1-second light exposure followed by 10 seconds of darkness was used due to their slower photo-response time.

Impedance and Capacitance measurement. The device architecture of FTO/NiOx/active material /GaIn device structure was used to measure capacitance and resistance of devices with and without light

exposure (GWINSTEK LCR-6200). The AC voltage perturbation was 10 mV under open-circuit conditions. Each frequency spectrum was measured ranging between 10 Hz and 200 kHz at a given constant incident power (100 mW/cm^2).

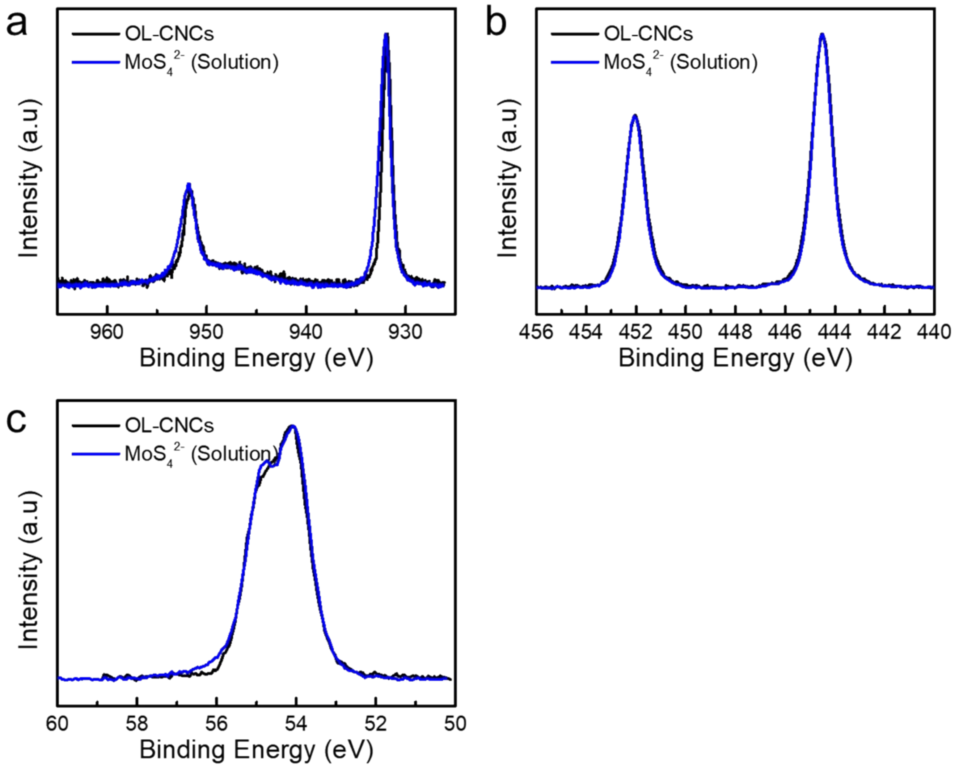

Figure S1. The core-level spectra of (a) Cu 2p, (b) In 3d, and (c) Se 3d for MoS_4^{2-}-treated CNCs in the solution phase and OL_CNCs.

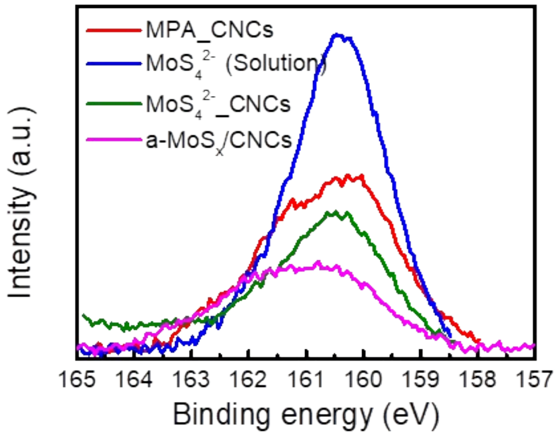

Figure S2. The core-level spectra of S 2p for CNCs with four different ligand systems.

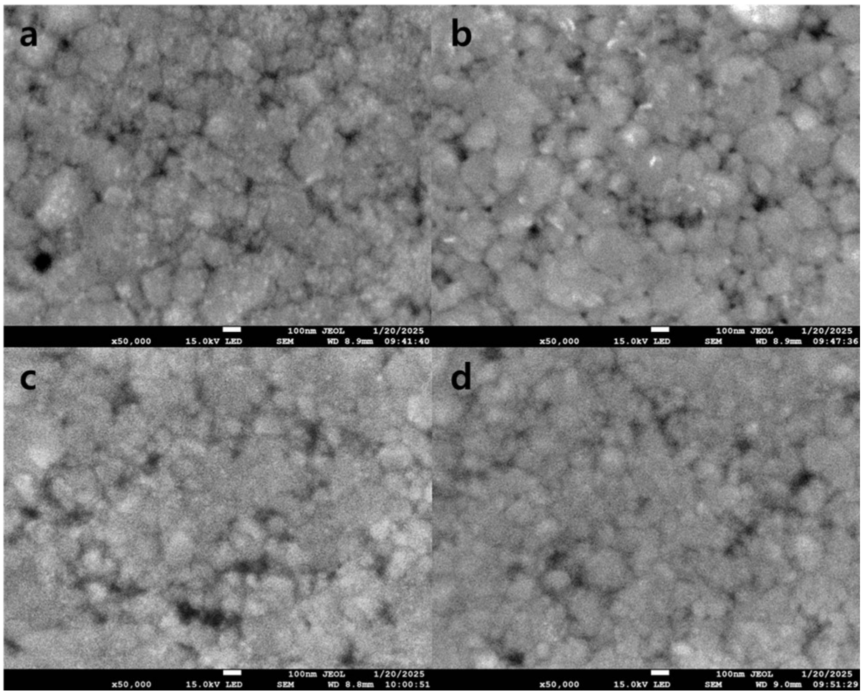

Figure S3. Top view of SEM images which is obtained from (a) OL_CNCs, (b) MPA_CNCs, (c) MoS_4^{2-}_CNCs and (d) a-MoS_x/CNCs.

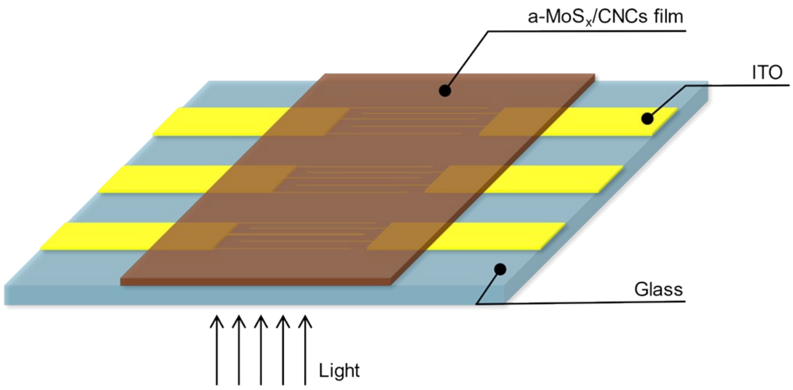

Figure S4. Illustration of the structure of lateral-type photodetector with a channel size of 600 μm × 40 μm which was used to measure photodetection performance.

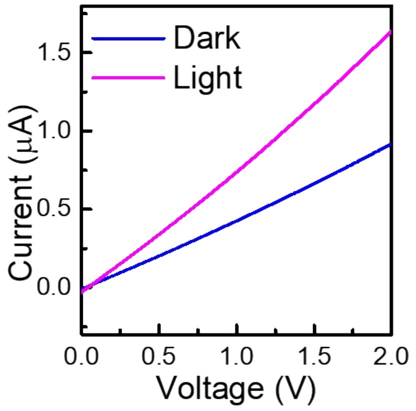

Figure S5. The static-state photocurrent measurement for MoS_4^{2-}_CNC-based photodetectors.

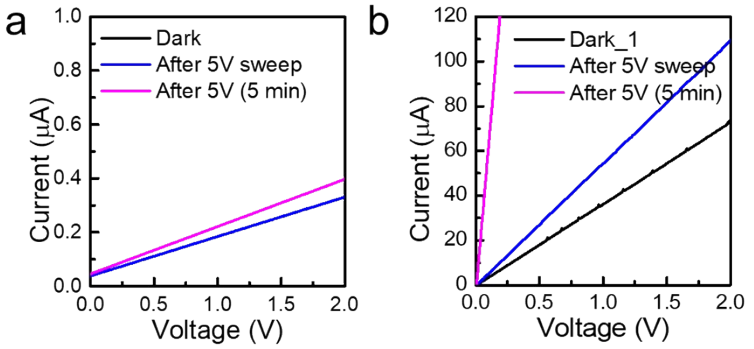

Figure S6. Voltage-current plots under dark conditions for (a) a-MoS_x/CNCs and (b) MPA _CNCs, based on operation cycles: the first dark cycle (Dark), the second dark cycle following a 0 to 5V sweep with AM 1.5 illumination (after the 5V sweep), and the third dark cycle after a constant voltage mode at 5V under AM 1.5 illumination for 5 minutes (after 5 minutes at 5V).

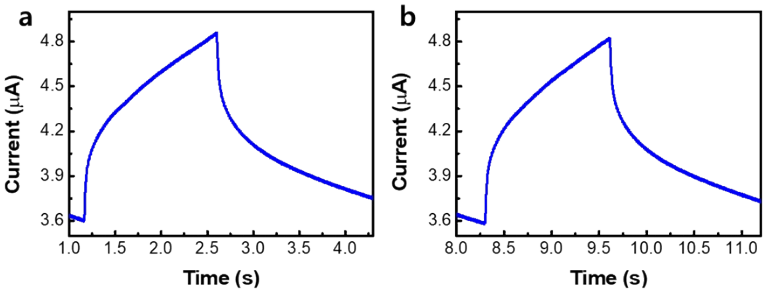

Figure S7. (a) Photodetection performance under AM1.5 illumination for a photodetector fabricated one month earlier. (b) Photodetection performance under AM1.5 illumination after 100 operational cycles. Photodetection behavior was measured 10 times separately for data storage, and the data in Figure S7b represent the performance during the 10th operational cycle.

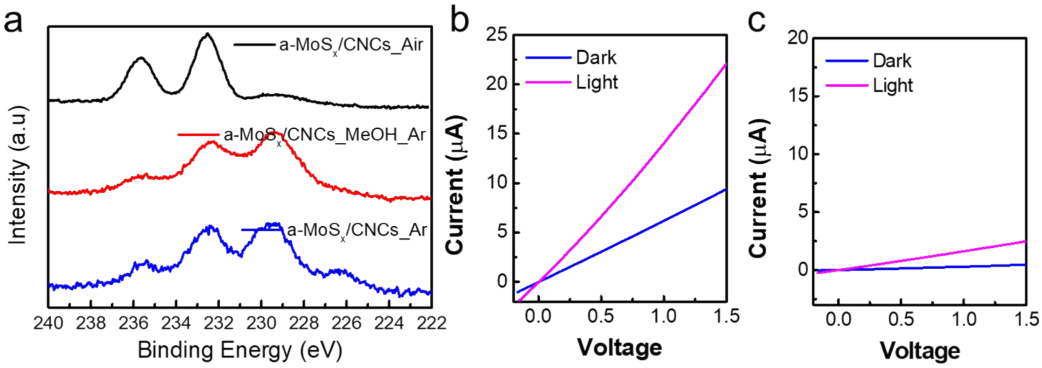

Figure S8. (a) The core-level spectra of Mo 3d for a-MoS$_x$/CNCs annealed at 250 °C under air condition (black solid line), with MeOH under inert condition (red solid line) and under inert condition after remove MeOH with soft annealing process (blue solid line), respectively. The static-state photocurrent measurements were conducted for (b) a-MoS_4^{2-}_CNC annealed under air conditions and (c) a-MoS_4^{2-}_CNC immediately annealed under Ar conditions without fully drying after spin coating.

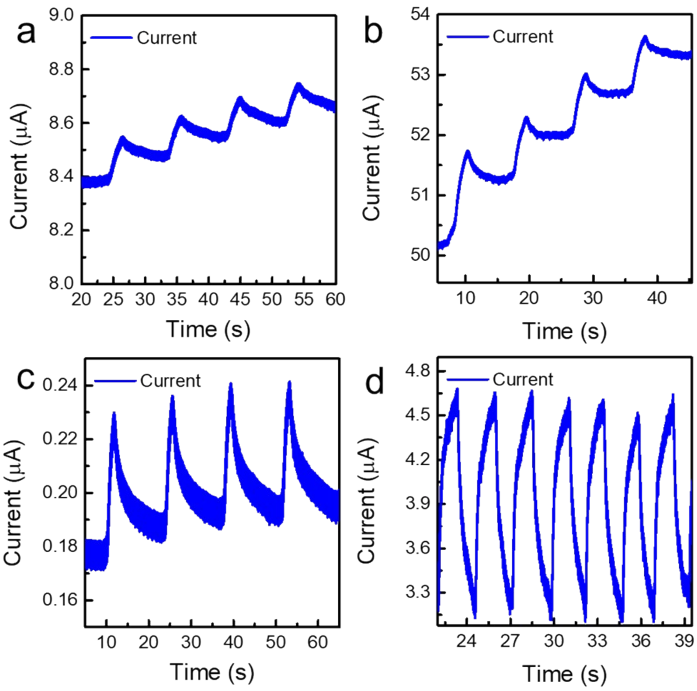

Figure S9. On-off response of photodetectors with (a) OL_CNCs, (b) MPA_CNCs, (c) MoS_4^{2-}_CNCs (solution), and (d) a-MoS$_x$/CNCs.

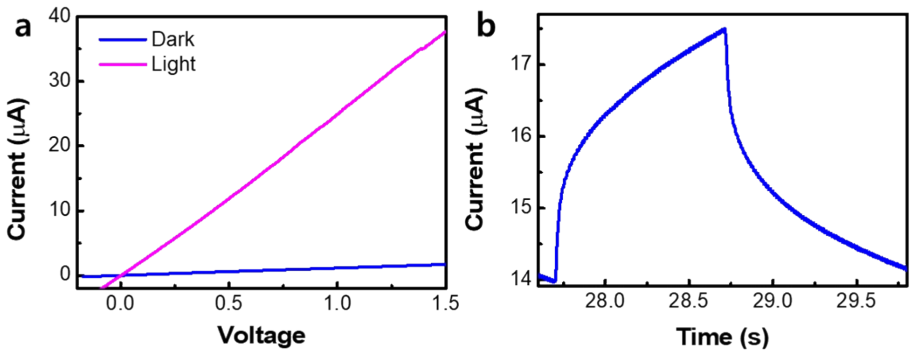

Figure S10. The performance of the a-MoS_x/CNCs photodetector evaluated under low-energy light irradiation.

Reference

(1) Tang, J.; Hinds, S.; Kelley, S. O.; Sargent, E. H. Synthesis of Colloidal CuGaSe2, CuInSe2, and Cu(InGa)Se2 Nanoparticles. *Chemistry of Materials* 2008, ***20*** (22), 6906-6910. DOI: 10.1021/cm801655w.

(2) Di Stasio, F.; Grim, J. Q.; Lesnyak, V.; Rastogi, P.; Manna, L.; Moreels, I.; Krahne, R. Single-Mode Lasing from Colloidal Water-Soluble CdSe/CdS Quantum Dot-in-Rods. *Small* 2015, ***11*** (11), 1328-1334. DOI: https://doi.org/10.1002/smll.201402527.

(3) Ban, H. W.; Park, S.; Jeong, H.; Gu, D. H.; Jo, S.; Park, S. H.; Park, J.; Son, J. S. Molybdenum and Tungsten Sulfide Ligands for Versatile Functionalization of All-Inorganic Nanocrystals. *The Journal of Physical Chemistry Letters* 2016, *7* (18), 3627-3635. DOI: 10.1021/acs.jpclett.6b01578.
